# Spiritual needs, practices and associated factors among patients with cancer at two teaching hospitals

**DOI:** 10.4102/phcfm.v17i1.5009

**Published:** 2025-09-30

**Authors:** Mpho Ratshikana, Daynia Ballot, Hellen Myezwa, Mary-Lou Galantino, Sonti Pilusa

**Affiliations:** 1 Wits Centre for Palliative Care, Faculty of Health Sciences, University of the Witwatersrand, Johannesburg, South Africa; 2 Department of Internal Medicine, Chris Hani Baragwanath Academic Hospital, Johannesburg, South Africa; 3 Department of Paediatrics and Child Health, Faculty of Health Sciences, University of the Witwatersrand, Johannesburg, South Africa; 4 Department of Physiotherapy, School of Therapeutic Sciences, University of the Witwatersrand, Johannesburg, South Africa; 5 Department of Integrative Health, School of Health Sciences, Stockton University, Galloway, United States; 6 Department of Physiotherapy, Centre for Clinical Epidemiology and Biostatistics, University of Pennsylvania, Philadelphia, United States

**Keywords:** spiritual needs, practices, spiritual care, cancer, palliative care, traditional health practitioners, ancestors

## Abstract

**Background:**

Patients diagnosed with cancer require holistic care that covers the physical, psychosocial and spiritual aspects of wellbeing.

**Aim:**

To describe the spiritual needs, practices, association between spirituality, using Traditional Health Practitioners (THPs), ancestral belief, with socio-demographic and clinical characteristics, and examine the association between spiritual interventions and spiritual characteristics.

**Setting:**

Palliative Care units at two tertiary hospitals in Johannesburg.

**Methods:**

An observational retrospective study used routinely collected data of patients older than 18 years, diagnosed with cancer, having complete records on spiritual questions, and enrolled between January 2021 and December 2023. Data were analysed using STATA V18.

**Results:**

Most participants (*n* = 2465) were female (70.5%), with a mean age of 53.6 (s.d.: 22.7). Half were unemployed, 40.1% married/partnered, and 54.4% living with HIV. Many participants were religious (94.1%) and spiritual (96.3%), Christian (84.3%), 11.3% consulted a TPHs, and 20.0% had ancestral beliefs. Most (94.7%) relied on their faith for comfort, their faith grew stronger (84.9%,) and 79.7% needed forgiveness. Receiving spiritual interventions was associated with the need for forgiveness, relying on faith for comfort and receiving support from the faith community.

**Conclusion:**

The study confirms that patients with cancer are spiritual and religious; some have ancestral beliefs, need forgiveness, and rely on their faith and faith communities to cope. The study further highlights the need for culturally relevant tools and interventions to address these needs.

**Contribution:**

The article highlights the unique spiritual beliefs and practices among patients with cancer that may influence planning for palliative care and cancer programmes.

## Introduction

Cancer is highlighted as a growing public health concern globally, as it negatively affects low- and middle-income countries (LMICs) disproportionately.^1^ South Africa, as a middle-income country, experiences a high burden of cancer, as well as high cancer mortality.^2^ The World Health Organization (WHO) developed a cancer strategy that encouraged countries to develop comprehensive cancer strategies to address the cancer burden and needs of patients.^3^ In line with this recommendation, the National Department of Health in South Africa developed the *National Cancer Strategic Framework, which recommends palliative care throughout the cancer continuum of care*.^4^ The National Policy Framework and Strategy for Palliative Care (Palliative Care Framework) recognises the need for holistic care, covering physical, social, emotional and spiritual care.^5^ In line with WHO recommendations, South Africa approved the *Traditional Health Practitioners Act 22 of 2007*, which has not yet been fully incorporated as part of the public health system.^6^ Evidence on the physical, social, emotional and spiritual aspects of cancer care is scarce in South Africa.

A diagnosis of a life-threatening illness causes distress, which can include spiritual distress.^7,8^ While there are many definitions for spirituality, spirituality in this study aligns with the consensus definition by Puchalski et al.^9^ According to Puchalski et al., spirituality is a human need and the way people seek meaning, purpose, transcendence and connections, expressed through beliefs, values and practices.^9^ While spirituality is different from religion, people can express their spirituality through religion.^9^ Spirituality is unique for each individual, and spiritual care provides a means of identifying these unique needs and developing a care plan that is specific to patients.^10^ Spiritual care is the process of assessing and addressing the spiritual needs of patients and families^11^ and is an important component of palliative cancer care, recommended by the South African national palliative care policy and other guidelines globally.^5,9,12,13^ According to these recommendations, health professionals can identify patients who have spiritual needs and who would benefit from spiritual care.

Most patients living with cancer in sub-Saharan Africa (SSA) use traditional, complementary and alternative medicine (TCAM).^14,15^ Patients living with a cancer diagnosis consult traditional healers for different reasons, such as not being covered by biomedical medicine, using traditional medicine, practising faith healing and praying.^16,17,18^ While research among patients living with cancer is increasing in SSA, such research is still limited in South Africa.^15^

Providing spiritual care and support for patients living with cancer is associated with better treatment outcomes, quality of life, improved coping, a source of hope and strength, pain and decision making,^19,20,21,22^ whereas a lack of spiritual care support is associated with poor quality of life and existential suffering, which negatively impacts treatment outcomes.^23,24^ Hence, the recommendation is that spiritual care should be incorporated as part of holistic person-centred care for people with cancer.^13,25^ Compared with high-income countries (HICs), research on the spiritual needs and practices of patients living with a cancer diagnosis in South Africa is limited and has not been incorporated as part of comprehensive cancer care, despite policy recommendations.^5,13,26^

This study aims to describe the spiritual and religious needs and practices of people living with cancer referred to palliative care units at two public tertiary hospitals in Johannesburg. The study specifically describes spiritual and religious needs and identifies common spiritual and religious practices among people living with cancer. The study further assesses the association between spirituality, having ancestral beliefs, consulting with the THP, socio-demographic and clinical characteristics. Lastly, the association between spiritual interventions and spiritual characteristics will be explored.

## Research methods and design

### Setting

The study was conducted at two academic hospitals in Johannesburg. Both hospitals provide cancer care, diagnosis, surgery and chemotherapy for adult patients living with cancer. The two hospitals jointly have a bed capacity of 4000 and have palliative care units that address the physical, social, emotional and spiritual aspects of patients living with cancer.

### Design

This was an observational retrospective study in which data were routinely collected at both hospitals. Patients living with a cancer diagnosis who are referred to palliative care are assessed by teams of doctors, nurses, social workers and spiritual counsellors and chaplains for physical, social, emotional and spiritual needs, followed by tailor-made interventions by the relevant team members. Spiritual counsellors/chaplains are volunteers who are trained in spirituality and chaplaincy in palliative care short course through the University of the Witwatersrand. Spiritual care interventions included spiritual counselling, facilitating reconciliations, family meetings and referral to spiritual leaders as appropriate and prayers.^21,27^ All the data were captured in the REDCap^28,29^ electronic database maintained at the Wits Centre for Palliative Care. Records of patients enrolled between January 2021 and December 2023 were extracted for patients who were older than 18 years, were diagnosed with cancer, and were able to respond to questions from the team. Records of patients who were recorded as unconscious and unable to be assessed and those with incomplete spiritual questions were excluded.

### Study data collection tools

Data were collected by teams of doctors, nurses, social workers, social auxiliary workers and spiritual counsellors via an electronic tool and captured via REDCap.^28,29^ The routine information collected included socio-demographic characteristics (age, sex, race, nationality, ethnic group, marital status, employment, level of education); clinical characteristics (diagnosis, staging, comorbidities); palliative outcome measures, which were determined using the Integrated Palliative Outcome Scale, a validated tool globally^30,31^; and, to determine spiritual needs, some questions from the Brief RCOPE^32^ and FICA,^33^ validated tools used internationally, were extracted. To address face validity, palliative care staff were consulted on the adapted questions that address ancestral and traditional beliefs, common practices and interventions, some of which are described by Puchalski et al. and Zuma et al.^27,34^ (Online Appendix 1: Supplement A).

### Data analysis

Descriptive statistics were used to summarise the characteristics of the study sample, and the results were presented via different summary statistics for continuous and categorical variables. For categorical variables, frequencies (*n*) and percentages (%) were used to describe the data. The means and standard deviations (s.d.s) were used for normally distributed data, whereas the medians and interquartile ranges were used for skewed data. For bivariate analysis, Chi-square tests were conducted to assess associations, and independent *t* tests were used to compare age. The level of significance was set at 5%.

### Ethical considerations

The study was approved by the University of the Witwatersrand Health Research Committee M240205; MED24-01-375, Charlotte Maxeke Johannesburg Academic Hospital (CMJAH) (No. GP_202403_059) and Chris Hani Baragwanath Academic Hospital (NHRD number: GP_202403_059). Ethical standards of the two institutional public tertiary hospitals were adhered to. Approval for the use of the deidentified dataset was obtained from management at both hospitals. Consent from participants was not required because the study was part of routine clinical care and retrospective, and the data were deidentified, which the ethics committee waived.

## Results

**FIGURE 1:**
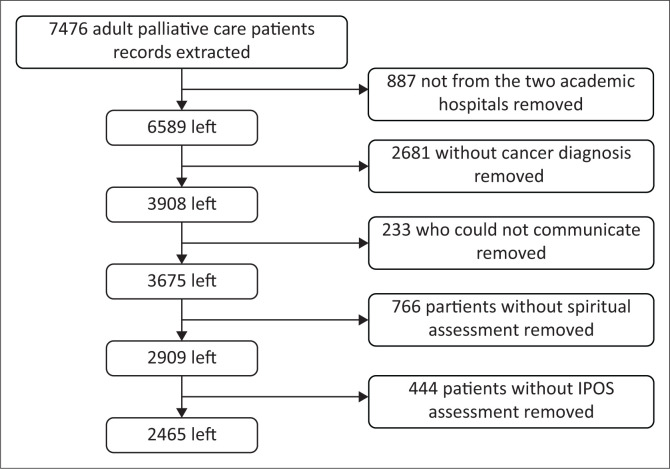
Flow chart of extracted records.

Of the 3908 records for patients living with a cancer diagnosis who were retrieved, only 2465 records with complete information on spiritual characteristics were included in the analysis (Figure 1).

### Socio-demographic characteristics of patients living with a cancer diagnosis at the two academic hospitals in Johannesburg

Most of the patients were black (81.8%), women (70.5%), with a mean age of 53.6 years. Half (51.9%) of the patients were unemployed, 78.2% had high school, technical high school and tertiary education, while 40.1% and 40.5% were married or partnered and single, respectively. Table 1 outlines the patients’ demographic profiles.

**TABLE 1:** Socio-demographic characteristics of adult patients living with a cancer diagnosis at the two academic hospitals (*N* = 2465).

Demographic characteristics	Mean	s.d.	Number	%
Age (years)	53.4	22.7	-	-
**Sex**				
Male	-	-	725	29.4
Female	-	-	1739	70.5
Missing	-	-	1	0.0
**Race**				
White people	-	-	248	10.1
Black people	-	-	2017	81.8
Mixed race people	-	-	132	5.4
Indian people	-	-	61	2.5
Asian Chinese/Japanese people	-	-	2	0.1
Other	-	-	4	0.2
Missing	-	-	1	0.0
**Marital status**				
Married/partnered	-	-	988	40.1
Widowed	-	-	294	11.9
Single	-	-	998	40.5
Divorced/separated	-	-	175	7.1
Refused to say	-	-	3	0.1
Missing	-	-	7	0.3
**Employment status**				
Employed full time	-	-	433	17.6
Employed part time/piece work	-	-	88	3.6
Informal employment/informal trading	-	-	56	2.3
Unemployed	-	-	1279	51.9
Retired	-	-	587	23.8
Missing	-	-	22	0.9
**Highest level of education**				
No formal education	-	-	104	4.2
Primary school	-	-	284	11.5
High School	-	-	1467	59.5
Technical school	-	-	197	8.0
Graduate – Tertiary education	-	-	264	10.7
Missing	-	-	149	6.0

s.d., standard deviation.

### Clinical characteristics of patients living with a cancer diagnosis at the two academic hospitals in Johannesburg

**FIGURE 2:**
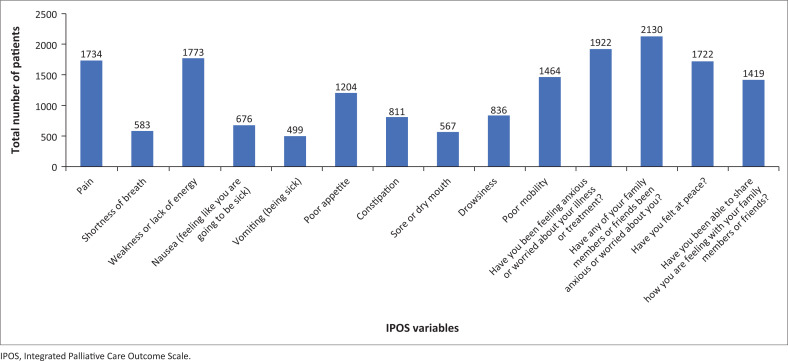
Baseline outcome measures using the Integrated Palliative Care Outcome Scale for patients living with a cancer diagnosis at the two hospitals in Johannesburg.

The most common cancers among the patients were breast (26.9%), cervix (22.2%) and gastro-intestinal, including hepatobiliary (20.2%) cancer. Lung and prostate cancer accounted for 6.9% and 5.7%, respectively. Almost half (54.4%) of the patients were living with HIV, 28.8% had hypertension and 8.4% had a diagnosis of diabetes. Common symptoms included pain, weakness/fatigue and poor mobility (see Figure 2). Most patients (78.0%) were worried about their disease, 86.4% reported family worry, while 69.9% felt at peace despite their diagnosis. Table 2 shows the clinical characteristics of the participants.

**TABLE 2:** Clinical characteristics of patients living with a cancer diagnosis at the two academic hospitals (*N* = 2465).

Clinical characteristics	Number	%
**Type of cancer**		
Breast	664	26.9
Cervix	546	22.2
GIT/Hepatobiliary	499	20.2
Lung	169	6.9
Prostrate	140	5.7
Other cancer	447	18.1
**HIV status**		
Negative	1062	43.0
Positive	1340	54.4
Missing	63	2.6
**TB diagnosis**		
No	1930	78.3
Yes	33	1.3
Missing	502	20.4
**High blood pressure**		
No	1684	68.3
Yes	710	28.8
Missing	71	2.9
**High blood sugar/diabetes**		
No	2187	88.7
Yes	206	8.4
Missing	72	2.9
**Other comorbidities**		
No	2170	88.0
Yes	229	9.3
Missing	66	2.7

### Spiritual and religious needs and characteristics of patients living with a cancer diagnosis at the two academic hospitals in Johannesburg

Almost all the participants agreed and strongly agreed that they were religious (94.1%), 96.3% were spiritual and 93.1% purportedly both spiritual and religious. Among the 494 with ancestral beliefs, 432 (87.4%) also reported having a Christian religion. Only 269 (11.3%) of the participants reported consulting a traditional healer/alternative medical practitioner (THP). Concerning the other spiritual and religious needs, 74.6% agreed and strongly agreed that they were looking for a stronger connection with God or Allah or Yahweh or ancestors, and 89.9% of the 494 who had ancestral beliefs were looking for a stronger connection with their ancestors. Most (94.7%) participants agreed and strongly agreed that their faith offered them comfort, whereas 84.9% indicated that their faith grew stronger after they became sick.

Only 279 (11.9%) participants consulted THPs, while 198 practised rituals. Of those who consulted THPs, half (53.5%) consulted THPs for prophecy, 19.0% because THPs understand the cause of the disease and 10.4% because THPs use herbs. The most common spiritual interventions provided by the spiritual counsellors were spiritual counselling (52.5%) and family meetings (25.6%).

### Associations between spirituality, consulting with the traditional healer practitioner, having ancestral beliefs and socio-demographic and clinical characteristics among patients living with a cancer

Being spiritual was associated with being female, unemployed, having a high school education, diagnosed with breast and cervical cancer, living with HIV and having a diabetes diagnosis. (see Table 3 and Table 4). Being religious was associated with being female, being black, and having female cancers. Consulting the THP was associated with younger age (< 50 years), being black, female, unemployed, single, having a high school education and having a cervical cancer diagnosis. Ancestral belief was associated with being black and living with HIV (see Table 3 and Table 4).

**TABLE 3:** Association between spirituality, having ancestral beliefs, consulting with the THP, being religious and socio-demographic characteristics among patients living with a cancer at the two academic hospitals in Johannesburg.

Demographic characteristics	Do you consider yourself spiritual?†			Do you believe in ancestors?†			Have you consulted with a traditional healer?†			Are you religious?†		
	*n*	%	*p*	*n*	%	*p*	*n*	%	*p*	*n*	%	*p*
*N*	2366	96.4	-	487	98.8	-	279	11.9	-	2242	93.6	-
**Sex**	-	-	**0.002**	-	-	0.955	-	-	**0.014**	-	-	**< 0.001**
Male	681	28.8	-	157	32.2	-	99	35.5	-	631	28.2	-
Female	1684	71.2	-	330	67.8	-	180	64.5	-	1610	71.8	-
**Race**	-	-	0.175	-	-	**< 0.001**	-	-	**< 0.001**	-	-	**< 0.001**
White people	232	9.8	-	0	0.0	-	0	0.0	-	205	9.1	-
Black people	1942	82.1	-	484	99.4	-	277	99.3	-	1847	82.4	-
Mixed race people	127	5.4	-	2	0.4	-	0	0.0	-	125	5.6	-
Asian/Indian people	65	2.7	-	1	0.2	-	2	0.7	-	64	2.9	-
**Marital status**	-	-	0.673	-	-	0.662	-	-	**0.013**	-	-	0.539
Married/partnered	943	40.0	-	188	38.8	-	118	42.3	-	892	39.9	-
Widowed	285	12.1	-	49	10.1	-	20	7.2	-	271	12.1	-
Single	961	40.7	-	219	45.2	-	127	45.5	-	916	41.0	-
Divorced/separated	170	7.2	-	29	6.0	-	13	4.7	-	155	6.9	-
Refused to say	0	0.0	-	0	0.0	-	1	0.4	-	0	0.0	-
**Employment status**	-	-	**0.021**	-	-	0.317	-	-	**< 0.001**	-	-	0.066
Employed	565	24.1	-	116	24.1	-	62	22.5	-	531	23.9	-
Unemployed	1218	51.9	-	268	55.6	-	169	61.5	-	1172	52.7	-
Retired	563	24.0	-	98	20.3	-	44	16.0	-	521	23.4	-
**Highest level of education**	-	-	**0.040**	-	-	0.887	-	-	**0.007**	-	-	**< 0.001**
No formal education	96	4.3	-	27	5.8	-	12	4.5	-	91	4.3	-
Primary school	274	12.3	-	64	13.8	-	31	11.7	-	263	12.5	-
High School	1414	63.4	-	315	68.0	-	191	71.8	-	1347	63.9	-
Technical school	196	8.8	-	27	5.8	-	10	3.8	-	184	8.7	-
Graduate – Tertiary education	249	11.2	-	30	6.5	-	22	8.3	-	223	10.6	-

Note: Significant *p*-value bolded.

† , yes.

**TABLE 4:** Association between spirituality, having ancestral beliefs, consulting with the THP, being religious and clinical characteristics among patients living with a cancer at the two academic hospitals in Johannesburg.

Clinical characteristics	Do you consider yourself spiritual?†			Do you believe in ancestors?†			Have you consulted with a traditional healer?†			Are you religious?†		
	*n*	%	*p*	*n*	%	*p*	*n*	%	*p*	*n*	%	*p*
*N*	2366	96.4	-	487	98.8	-	279	11.9	-	2242	93.6	-
**Type of cancer**	-	-	**0.027**	-	-	0.082	-	-	**0.016**	-	-	**0.035**
Breast	631	26.7	-	112	23.0	-	52	18.6	-	607	27.1	-
Cervix	532	22.5	-	126	25.9	-	81	29.0	-	503	22.4	-
GIT/Hepatobiliary	476	20.1	-	94	19.3	-	55	19.7	-	458	20.4	-
Lung	160	6.8	-	29	6.0	-	18	6.5	-	149	6.6	-
Prostrate	130	5.5	-	33	6.8	-	16	5.7	-	123	5.5	-
Other cancer	437	18.5	-	93	19.1	-	57	20.4	-	402	17.9	-
**HIV status**	-	-	**0.002**	-	-	**< 0.001**	-	-	0.055	-	-	0.513
Positive	1298	55.1	-	288	59.3	-	170	61.4	-	1219	54.6	-
Negative	1011	42.9	-	188	38.7	-	104	37.5	-	968	43.3	-
Unknown	47	2.0	-	10	2.1	-	3	1.1	-	47	2.1	-
**TB diagnosis**	-	-	0.439	-	-	0.972	-	-	0.396	-	-	**0.018**
No	1861	97.3	-	391	97.3	-	228	96.6	-	1769	97.5	-
Yes	33	1.7	-	9	2.2	-	6	2.5	-	31	1.7	-
Unknown	19	1.0	-	2	0.5	-	2	0.8	-	15	0.8	-
**High blood pressure**	-	-	0.425	-	-	0.246	-	-	0.232	-	-	0.426
No	1618	70.2	-	342	71.5	-	202	73.5	-	1534	70.3	-
Yes	688	29.8	-	136	28.5	-	73	26.5	-	649	29.7	-
**High blood sugar/diabetes**	-	-	0.271	-	-	0.370	-	-	**0.006**	-	-	0.625
No	2105	91.3	-	443	92.9	-	264	96.0	-	1997	91.5	-
Yes	200	8.7	-	34	7.1	-	11	4.0	-	185	8.5	-
**Other comorbidities**	-	-	0.164	-	-	0.404	-	-	0.186	-	-	0.613
No	2094	90.6	-	441	92.5	-	255	92.7	-	1982	90.6	-
Yes	217	9.4	-	36	7.5	-	20	7.3	-	206	9.4	-

Note: Significant *p*-value bolded.

† , yes.

### Associations between spiritual interventions and spiritual characteristics among patients living with cancer

The associations between receiving spiritual interventions and spiritual characteristics are shown in Table 5. Receiving spiritual intervention is not influenced by the type of religion, whether the individual is spiritual or religious, belief in ancestors or consulting the THP. Only participants who felt that their faith offered them comfort, those who felt supported by their faith communities, those who were asking for forgiveness and those who practised rituals were more likely to receive spiritual counselling. The participants who felt abandoned by their God or Allah or Yahweh or ancestors were less likely to receive spiritual counselling.

**TABLE 5:** Associations between spiritual characteristics and interventions for patients living with cancer at the two academic hospitals in Johannesburg.

Spiritual characteristics	Spiritual interventions										
	Spiritual counselling		Family meetings		Telephonic counselling		Bereavement		Total		Test
	*n*	%	*n*	%	*n*	%	*n*	%	*n*	%	
**Do you believe in ancestors?**											0.051
No	1	0.1	4	0.6	0	0.0	0	0.0	5	1.2	-
Yes	293	22.7	104	16.5	6	27.3	10	19.2	413	98.8	-
**My faith/spiritual belief is of comfort to me**											< 0.001
No	34	2.6	43	6.8	1	4.5	1	1.9	79	4.0	-
Yes	1253	96.9	584	92.6	21	95.5	51	98.1	1909	96.0	-
**I have been wondering whether God/Allah/Yahweh/Ancestors have abandoned me**											< 0.001
No	1131	87.5	538	85.3	20	90.9	35	67.3	1724	86.6	-
Yes	159	12.3	90	14.3	2	9.1	16	30.8	267	13.4	-
**I feel supported by my faith community**											< 0.001
No	256	19.8	191	30.3	7	31.8	15	28.8	469	23.7	-
Yes	1026	79.4	435	68.9	15	68.2	35	67.3	1511	76.3	-
**I have been asking people and God/Allah/Yahweh/Ancestors for forgiveness**											< 0.001
No	162	12.5	144	22.8	11	50.0	22	42.3	339	17.1	-
Yes	1125	87.0	478	75.8	11	50.0	29	55.8	1643	82.9	-
**Do you practice any rituals?**											0.010
Yes	126	78.3	45	71.4	2	66.7	3	30.0	176	77.2	-
No	30	18.6	15	23.8	1	33.3	6	60.0	52	22.8	-

## Discussion

This study revealed that most patients living with cancer have spiritual needs. Similar to previous studies, patients living with cancer desire forgiveness and closer connections. These are both spiritual and religiously oriented, and patients use their faith to navigate the cancer disease journey.^19,22,35^ In a systematic review, Balboni et al. highlighted that patients diagnosed with serious illnesses, including cancer, have spiritual needs that must be addressed as part of their holistic care.^36^

Transcendence and connections with self and others are important dimensions of spirituality among patients living with cancer, as reported elsewhere.^8,9,19,37^ In this study, most patients expressed the need for closer connections with higher power, God and ancestors. In addition, the patients embraced support from their faith communities.^19,34^ Masola explained that for African patients diagnosed with chronic illnesses, restoring relationships with the supernatural may be part of the healing process for some patients.^38^

Like in previous studies, some patients in this study not only relied on their faith in coping with their illness but also consulted THPs.^19,22,35,39^ The use of THPs in this research is, however, lower than what was previously reported.^21,39,40^ Low reporting rates for THP use have been associated with easy access to health facilities.^39^ However, on the other hand, patients do not disclose the use of THP for fear of being discriminated against by health professionals, or they do not feel the need to share the information.^35,36,37^ In a study conducted in Langa, Cape Town, Hughes (2015) reported that participants cited accessibility and affordability as reasons for using THPs.^41^

Of note is that most of the research conducted on THP use among patients living with cancer has been conducted mainly in Nigeria, Ethiopia and Ghana.^14^ This study fills this knowledge gap.

Previous research highlighted that patients living with cancer combine biomedical medicine and use THPs for different reasons. Patients with cancer use THPs because they believe that the disease has both physical and spiritual causes, that THPs give them hope, that they can connect them to ancestors, and that they can prophesy and have supernatural powers for faith healing.^42,43,44,45^ Efforts to integrate traditional health and biomedical medicine have been unsuccessful, despite recommendations globally and by the *South African Traditional Health Practitioners Act*.^6^

According to some African scholars, there seems to be a link between belief in God, or the higher being, ancestors (who protect, bless and can curse descendants) and the use of THPs, who believe in witchcraft and the practice of rituals.^42,46,47,48^ This might explain why some patients in this study reported having both ancestral and Christian beliefs. Research on the role of ancestors as part of religious and spiritual care in palliative and cancer care is limited. The need for forgiveness from ancestors among study participants may be a way of expressing the need to appease ancestors to avoid misfortunes, which can manifest as illness.^42,46^

The spiritual care interventions used in this study included spiritual counselling, facilitating reconciliations, family meetings and referral to spiritual leaders as appropriate and prayers.^21,27^ Receiving spiritual counselling was not influenced by the type of religion, being spiritual or religious, having a belief in ancestors or consulting the THPs in this study, confirming that providing spiritual care can address diverse palliative care patients, irrespective of their belief system, in line with previous recommendations.^48^ Receiving spiritual interventions was associated with having a spiritual need and relying on faith for support and coping, highlighting the importance of spiritual assessment to identify needs that guide spiritual care interventions relevant to patients’ needs. Patients who felt abandoned were less likely to receive spiritual support. This might be because they experience hidden loneliness and suffering that is not visible to others, as explained elsewhere.^49^ This highlights the need for trained spiritual care counsellors and chaplains who can build trust and rapport to engage patients at a deeper level, because abandonment, being part of a religious struggle, has been associated with poor treatment outcomes.^50^

Like in previous studies, spiritual and religious beliefs can be associated with some socio-demographic and clinical characteristics. Being female, unemployed, black and having low socio-economic status is associated with spiritual and religious beliefs and the use of THPs,^43,51,52^ whereas breast cancer and cervical cancer are associated with being spiritual, religious and consulting THPs. More research to determine the associations between socio-demographic and clinical characteristics and spiritual, religious, ancestral and traditional beliefs is recommended.

### Strengths and limitations

Limitations of the study include its retrospective nature, which may have introduced bias. The study participants are predominantly black people in urban settings and are mostly Christian, with missing values for some of the variables, particularly in response to ‘Do you believe in ancestors’ and ‘Do you practice rituals?’ The strengths of this study include its large sample size and use of validated tools.

### Implications and recommendations

More research is needed to understand spiritual needs and develop relevant tools and interventions for the current setting. Policymakers and clinicians should consider integrating spiritual care as a component of cancer care across the continuum of care. Research to understand the use of THPs among patients living with cancer in the current setting is recommended. Culturally sensitive spiritual care tools that embrace the use of THPs and incorporate ancestral beliefs and interventions that capture rituals specific to the current population should be prioritised. Research investigating the reasons for less acceptance of spiritual counselling by patients who express abandonment by God or Allah or ancestors is recommended.

## Conclusion

This study highlights that patients living with cancer are spiritual and religious. Patients have ancestral beliefs, need forgiveness and connections with God, higher beings and ancestors, rely on their faith and faith communities to cope, and some use THPs for distinct reasons. Receiving spiritual interventions was positively associated with being ritualistic and providing comfort, whereas spiritual care ignites feelings of abandonment and the need for forgiveness. Research should explore specifically what patients are seeking forgiveness for in the context of their lived experience and determine rituals practised and THPs used among this population.

## References

[1] Shah SC, Kayamba V, Peek RM, Heimburger D. Cancer control in low- and middle-income countries: Is it time to consider screening? J Global Oncol. 2019;(5):1–8. 10.1200/JGO.18.00200 PMC6452918 30908147

[2] Sharma R, Aashima, Nanda M, et al. Mapping cancer in Africa: A comprehensive and comparable characterization of 34 cancer types using estimates from GLOBOCAN 2020. Front Public Health. 2022;10:839835. 10.3389/fpubh.2022.839835 35548083 PMC9082420

[3] WHO Cancer Report [homepage on the Internet]. Available from: https://www.quotidianosanita.it/allegati/allegato4849716.pdf.

[4] National Department of Health [Internet]. [cited 2024 Oct 23]. Available from: https://www.health.gov.za/wp-content/uploads/2020/11/national-cancer-strategic-framework-2017-2022-min.pdf.

[5] National Department of Health [Internet]. [cited 2024 Oct 23]. Available from: https://www.health.gov.za/wp-content/uploads/2020/11/NationalPolicyFrameworkandStrategyonPalliativeCare20172022.pdf.

[6] Traditional Health Practitioners Act 22 of 2007 | South African Government [homepage on the Internet]. [cited 2025 Apr 13]. Available from: https://www.gov.za/documents/traditional-health-practitioners-act.

[7] Bai M, Lazenby M. A systematic review of associations between spiritual well-being and quality of life at the scale and factor levels in studies among patients with cancer. J Palliat Med. 2015;18(3):286–298. 10.1089/jpm.2014.0189 25303461 PMC4348086

[8] Puchalski CM, King SDW, Ferrell BR. Spiritual considerations. Hematol Oncol Clin N A. 2018;32(3):505–517. 10.1016/j.hoc.2018.01.011 29729785

[9] Puchalski C, Ferrell B, Virani R, et al. Improving the quality of spiritual care as a dimension of palliative care: The report of the consensus conference. J Palliat Med. 2009;12(10):885–904. 10.1089/jpm.2009.0142 19807235

[10] Fitch MI, Bartlett R. Patient perspectives about spirituality and spiritual care. Asia-Pacific J Oncol Nurs. 2019;6(2):111–121. 10.4103/apjon.apjon_62_18 PMC6371668 30931354

[11] Tavares AP, Martins H, Pinto S, Caldeira S, Pontífice Sousa P, Rodgers B. Spiritual comfort, spiritual support, and spiritual care: A simultaneous concept analysis. Nurs Forum. 2022;57(6):1559–1566. 10.1111/nuf.12845 36448491 PMC10099816

[12] Ferrell BR, Twaddle ML, Melnick A, Meier DE. National consensus project clinical practice guidelines for quality palliative care guidelines, 4th Edition. J Palliat Med. 2018;21(12):1684–1689. 10.1089/jpm.2018.0431 30179523

[13] Selman LE, Harding-Swale R, Agupio G, et al. Spiritual care recommendations for people receiving palliative care in sub-Saharan Africa: With special reference to South Africa and Uganda [homepage on the Internet]. London: Cicely Saunders International; 2010 [cited 2024 Nov 30]. Available from: https://www.kcl.ac.uk/lsm/research/divisions/cicelysaunders/attachments/Spiritual-care-Africa-Full-report.pdf.

[14] James PB, Wardle J, Steel A, Adams J. Traditional, complementary and alternative medicine use in Sub-Saharan Africa: A systematic review. BMJ Glob Health. 2018;3(5):e000895. 10.1136/bmjgh-2018-000895 PMC6231111 30483405

[15] James PB, Asiimwe JB, Wardle J, Mwaka AD, Kasilo OMJ. African culture, traditional medicine, and cancer care. Lancet Oncol. 2022;23(6):705–706. 10.1016/S1470-2045(22)00157-7 35550271

[16] Asuzu CC, Akin-Odanye EO, Asuzu MC, Holland J. A socio-cultural study of traditional healers role in African health care. Infect Agents Cancer. 2019;14(1):15. 10.1186/s13027-019-0232-y PMC6585125 31249608

[17] Ezeome ER. Delays in presentation and treatment of breast cancer in Enugu, Nigeria. Niger J Clin Pract. 2010;13(3):311–316. 20857792

[18] Mwaka AD, Abbo C, Kinengyere AA. Traditional and complementary medicine use among adult cancer patients undergoing conventional treatment in sub-Saharan Africa: A scoping review on the use, safety and risks. Cancer Manage Res. 2020;12:3699–3712. 10.2147/CMAR.S251975 PMC7246319 32547206

[19] Vallurupalli M, Lauderdale K, Balboni MJ, et al. The role of spirituality and religious coping in the quality of life of patients with advanced cancer receiving palliative radiation therapy. J Support Oncol. 2012;10(2):81–87. 10.1016/j.suponc.2011.09.003 22088828 PMC3391969

[20] Chen J, Lin Y, Yan J, Wu Y, Hu R. The effects of spiritual care on quality of life and spiritual well-being among patients with terminal illness: A systematic review. Palliat Med. 2018;32(7):1167–1179. 10.1016/j.suponc.2011.09.003 29708010

[21] Ratshikana-Moloko M, Ayeni O, Tsitsi JM, et al. Spiritual care, pain reduction, and preferred place of death among advanced cancer patients in Soweto, South Africa. J Pain Sympt Manage. 2020;60(1):37–47. 10.1016/j.jpainsymman.2020.01.019 PMC7311268 32045675

[22] VanderWeele TJ. Effects of religious service attendance and religious importance on depression: Examining the meta-analytic evidence. Int J Psychol Religion. 2021;31(1):21–26. 10.1080/10508619.2020.1748932

[23] Pearce MJ, Coan AD, Herndon JE, Koenig HG, Abernethy AP. Unmet spiritual care needs impact emotional and spiritual well-being in advanced cancer patients. Support Care Cancer. 2012;20(10):2269–2276. 10.1007/s00520-011-1335-1 22124529

[24] Astrow AB, Wexler A, Texeira K, He MK, Sulmasy DP. Is failure to meet spiritual needs associated with cancer patients’ perceptions of quality of care and their satisfaction with care? J Clin Oncol. 2007;25(36):5753–5757. 10.1200/JCO.2007.12.4362 18089871

[25] Crawford GB, Dzierżanowski T, Hauser K, et al. Care of the adult cancer patient at the end of life: ESMO clinical practice guidelines. ESMO Open. 2021;6(4):100225. 10.1016/j.esmoop.2021.100225 34474810 PMC8411064

[26] Osman H, Shrestha S, Temin S, et al. Palliative care in the global setting: ASCO resource-stratified practice guideline. J Global Oncol. 2018;(4):1–24. 10.1200/JGO.18.00026 PMC6223509 30085844

[27] Puchalski CM, Sbrana A, Ferrell B, et al. Interprofessional spiritual care in oncology: A literature review. ESMO Open. 2019;4(1):e000465. 10.1136/esmoopen-2018-000465 30962955 PMC6435249

[28] Harris PA, Taylor R, Minor BL, et al. The REDCap consortium: Building an international community of software platform partners. J Biomed Inform. 2019;95:103208. 10.1016/j.jbi.2019.103208 31078660 PMC7254481

[29] Harris PA, Taylor R, Thielke R, Payne J, Gonzalez N, Conde JG. Research electronic data capture (REDCap) – A metadata-driven methodology and workflow process for providing translational research informatics support. J Biomed Inform. 2009;42(2):377–381. 10.1016/j.jbi.2008.08.010 18929686 PMC2700030

[30] Schildmann EK, Groeneveld EI, Denzel J, Brown A, Bernhardt F, Bailey K, et al. Discovering the hidden benefits of cognitive interviewing in two languages: The first phase of a validation study of the Integrated Palliative care Outcome Scale. Palliat Med. 2016 Jun;30(6):599–610. 26415736 10.1177/0269216315608348 PMC4873725

[31] Palliative care Outcome Scale (POS) – POS downloads [homepage on the Internet]. [cited 2025 Jun 12]. Available from: https://pos-pal.org/maix/pos-downloads.php

[32] Pargament K, Feuille M, Burdzy D. The brief RCOPE: Current psychometric status of a short measure of religious coping. Religions. 2011;2(1):51–76. 10.3390/rel2010051

[33] Borneman T, Ferrell B, Puchalski CM. Evaluation of the FICA tool for spiritual assessment. J Pain Sympt Manage. 2010;40(2):163–173. 10.1016/j.jpainsymman.2009.12.019 20619602

[34] Zuma T, Wight D, Rochat T, Moshabela M. The role of traditional health practitioners in Rural KwaZulu-Natal, South Africa: Generic or mode specific? BMC Complement Altern Med. 2016;16(1):304. 10.1186/s12906-016-1293-8 27549895 PMC4994274

[35] Cheng Q, Xu X, Liu X, Mao T, Chen Y. Spiritual needs and their associated factors among cancer patients in China: A cross-sectional study. Support Care Cancer. 2018;26(10):3405–3412. 10.1007/s00520-018-4119-z 29663138

[36] Balboni TA, VanderWeele TJ, Doan-Soares SD, et al. Spirituality in serious illness and health. JAMA. 2022;328(2):184. 10.1001/jama.2022.11086 35819420

[37] Cheng L, Chen H, Lin L, Li H, Zhang F. Spiritual needs of older adults with cancer: A modified concept analysis. Asia Pac J Oncol Nurs. 2023;10(11):100288. 10.1016/j.apjon.2023.100288 38023729 PMC10661515

[38] Masola NJ, Sigida ST. Chronic diseases and medical pluralism: The case of faith healers in Limpopo, South Africa. Indilinga Afr J Indigenous Knowl Syst. 2021;20(2):194–208.

[39] Caldeira S, Carvalho EC, Vieira M. Spiritual distress-proposing a new definition and defining characteristics. Int J Nurs Knowl. 2013;24(2):77–84. 10.1111/j.2047-3095.2013.01234.x 23465219

[40] Adedini SA, Sello M, Thaele D, Madhi SA. Patterns of healthcare utilisation and barriers affecting access to child healthcare services in low-income urban South African settings [homepage on the Internet]. [cited 2025 Mar 18]. Available from: https://scielo.org.za/scielo.php?pid=S1999-76712020000100004&script=sci_abstract

[41] Hughes GD, Aboyade OM, Beauclair R, Mbamalu ON, Puoane TR. Characterizing herbal medicine use for noncommunicable diseases in urban South Africa. Evidence-Based Complement Alternat Med. 2015;2015(1):736074. 10.1155/2015/736074 PMC4629029 26557865

[42] Asiimwe JB, Nagendrappa PB, Atukunda EC, et al. The meaning of caring for patients with cancer among traditional medicine practitioners in Uganda: A grounded theory approach. PLOS Glob Public Health. 2023;3(7):e0001764. 10.1371/journal.pgph.0001764 37459297 PMC10351711

[43] Okyere Asante PG, Tuck CZ, Atobrah D. Medical pluralism, healthcare utilization and patient wellbeing: The case of Akan cancer patients in Ghana. Int J Qual Stud Health Well-Being. 2023;18(1):2238994. 10.1080/17482631.2023.2238994 37490583 PMC10392249

[44] Ong’udi M, Mutai P, Weru I. Study of the use of complementary and alternative medicine by cancer patients at Kenyatta National Hospital, Nairobi, Kenya. J Oncol Pharm Pract. 2019;25(4):918–928. 10.1177/1078155218805543 30319064

[45] Edwards Stephen. The role of ancestors in healing [homepage on the Internet]. Available from: https://hdl.handle.net/10520/EJC61564

[46] White P. The concept of diseases and health care in African traditional religion in Ghana. HTS Teologiese Studies/Theol Stud. 2015;71(3):1–7. 10.4102/hts.v71i3.2762

[47] Pew Research. Faith among Black Americans [homepage on the Internet]. Available from: https://www.pewresearch.org/religion/2021/02/16/faith-among-black-americans/

[48] Wierstra IR, Liefbroer AI, Post L, Tromp T, Körver J. Addressing spiritual needs in palliative care: Proposal for a narrative and interfaith spiritual care intervention for chaplaincy. J Health Care Chaplaincy. 2023;29(1):64–77. 10.1080/08854726.2021.2015055 34923933

[49] Quinn B. Making sense of pain and loss: Searching for meaning while living with cancer. Cancer Nurs Pract. 2018;17(5):29–36. 10.7748/cnp.2018.e1521

[50] Canada AL, Murphy PE, Stein K, Alcaraz KI, Leach CR, Fitchett G. Assessing the impact of religious resources and struggle on well-being: A report from the American Cancer Society’s Study of Cancer Survivors-I. J Cancer Surviv. 2023;17(2):360–369. 10.1007/s11764-022-01226-8 35726114 PMC10084782

[51] Thabede D. The African worldview as the basis of practice in the helping professions. Soc Work. 2014;44(3):1–13. 10.15270/44-3-237

[52] Farooqui M, Hassali MA, Shatar AKA, et al. Use of complementary and alternative medicines among Malaysian cancer patients: A descriptive study. J Trad Complement Med. 2016;6(4):321–326. 10.1016/j.jtcme.2014.12.008 PMC5067849 27774413

